# Exploring the global prevalence of mood and anxiety disorders in LGBTIQ+ people: A systematic review

**DOI:** 10.3389/fpsyt.2025.1662265

**Published:** 2025-12-04

**Authors:** Sarah Johnson, Mariia Bogdanova, Laith Alexander, Paul R. A. Stokes

**Affiliations:** Centre for Affective Disorders, Department of Psychological Medicine, Institute of Psychiatry, Psychology & Neuroscience, King’s College London, London, United Kingdom

**Keywords:** LGBTIQ+, mood disorder, anxiety disorder, global prevalence, systematic review

## Abstract

**Background:**

The prevalence of mood and anxiety disorders in LGBTIQ+ individuals (lesbian, gay, bisexual, transgender, intersex, queer, and other sexual/gender minorities) is not well understood. Studies suggest that LGBTIQ+ people may have higher rates of mood and anxiety disorders, potentially influenced by societal acceptance.

**Aims:**

This systematic review aims to examine the prevalence of depressive disorders (DD), bipolar disorders (BD), and anxiety disorders in LGBTIQ+ populations and explore potential associations with societal acceptance in different global regions.

**Methods:**

A systematic search of PubMed, Embase, APA PsychInfo, and citations from 1990–2022 identified studies reporting on the prevalence of DD, BD, and anxiety disorders among LGBTIQ+ people. These rates were compared to societal acceptance, using the Williams’ Institute Global Acceptance Index, and to general population rates. Study quality was assessed with the National Institute of Health checklist.

**Results:**

123 studies from 31 countries were included, with 116 rated as good quality. Individual study sample sizes ranged from 15 to over 254,462,596. Mean prevalence rates in LGBTIQ+ populations from these studies was 35.3% for depressive disorders, 5.6% for bipolar disorders, and 34.3% for anxiety disorders. A significant correlation was found between societal acceptance and depressive and anxiety disorder prevalence rates in North American LGBTIQ+ populations.

**Conclusions:**

This study found that LGBTIQ+ people experience markedly higher rates of mood and anxiety disorders compared to the general population, with societal acceptance correlating with these rates in North America. Further research is needed, particularly for underrepresented groups such as nonbinary individuals and those identifying as pansexual, asexual, or genderqueer.

**Systematic review registration:**

www.crd.york.ac.uk/prospero/, identifier CRD42022320324.

## Introduction

The prevalence and impact of affective disorders in lesbian, gay, bisexual, transgender, intersex, queer and people with other sexual/gender identities (LGBTIQ+) is not well understood, but trends in the evidence seem to indicate that LGBTIQ+ people may be more likely to be diagnosed with a mood or anxiety disorder. A meta-analysis by Meyer (1) found that lesbian, gay, and bisexual males and females were twice as likely to receive a mood or anxiety disorder diagnosis compared to heterosexuals. In the developed world, rates of mood disorders in LGBTIQ+ populations range from 17.1% (2) to 21.4% (3). The evidence in the developing world is even more striking, with rates of depression in LGBTIQ+ people ranging from 19.1% (4) to 56% (5).

The stressors that LGBTIQ+ people experience may be associated with, or exacerbate the symptomology, of depressive disorders (DD), bipolar disorders (BD), or anxiety disorders. A report by the LGBTIQ+ organisation Stonewall (6) found that one in five sexual/gender minority youths in the UK had experienced not only social isolation, which in itself is linked to the development of depression (7), but also experienced hate crimes. This figure rises to two in five for transgender individuals. Suicide rates are also elevated, with some studies reporting a four times increased risk of LGBTIQ+ youth suicides compared to heterosexual controls (8). Additionally, non-suicidal self-injury is more apparent in LGBTIQ+ people, with lifetime prevalence rates of 29.7% and 46.7% in sexual and gender minorities respectively, relative to 14.6% in heterosexual and/or cisgendered individuals (9).

The severity of the environmental stressors experienced by LGBTIQ+ people may relate to societal acceptance. A lack of societal acceptance could create a hostile, stigmatised and prejudiced environment, and the accompanying social, environmental and minority stressors are known to increase the risk of mental illness (1). This varies between countries and can be measured using the Williams’ Institute’s Global Acceptance Index (GAI) (10) which provides a quantitative measure of the societal acceptance of LGBTIQ+ people.

To date, previous reviews have focused on affective disorders in the context of co-morbidities (10, 11), or specific sub-groups (such as gay or lesbian individuals) of the LGBTIQ+ population. No systematic reviews to our knowledge have compared prevalence rates across the LGBTIQ+ spectrum, nor have they explored whether prevalence rates correlate with the degree of public acceptance. The primary aim of this systematic review was to investigate the prevalence of depressive disorders, bipolar disorders, and anxiety disorders in the LGBTIQ+ population across countries. This systematic review aimed to compare prevalence rates of mood and anxiety disorders between regions and to explore the relationship between prevalence rates and public acceptance and equality of LGBTIQ+ people.

## Methods

### Protocol

The study protocol was submitted to the NIHR PROSPERO International prospective register of systematic reviews on 1^st^ April 2022 and registered on 25^th^ July 2022 (PROSPERO registration number: CRD42022320324). We followed the PRISMA 2020 reporting guideline (12), and the completed PRISMA checklist is presented in **Supplementary Table 1**.

### Eligibility criteria

Inclusion and exclusion criteria for articles were determined prior to database searching. Studies were required to have investigated one (or more) of depressive disorders, bipolar disorders, and/or anxiety disorders in LGBTIQ+ populations. If participants had received a diagnosis of another primary psychiatric condition, the study was excluded. Diagnostic tools or measures were required to have been implemented in accordance with either DSM or ICD criteria. Studies exploring mood disorders in heterosexual and cisgendered individuals was included only if LGBTIQ+ populations were measured adjunctively as comparators. Studies including participants identifying as males who have sex with males, but not necessarily as gay or bisexual males, were included in this review as this group is often included in the “+” part of LGBTIQ +. There was no stipulated limitation on study settings or the age range of participants.

Cross-sectional, longitudinal, cohort, and retrospective chart reviews were included in the review while case studies and systematic reviews were excluded. Records were excluded from the review if English translations were unavailable, if studies were not peer-reviewed, or if studies focused primarily on potential mediators (such as HIV, minority stress, abuse, and COVID-19) that could have direct impact on prevalence rates of affective disorders. Moreover, studies were excluded if they did not include mood and anxiety disorder prevalence measures or odds ratios.

### Literature search

Two reviewers (SJ and OP) conducted the database searches independently between 1^st^ and 23^rd^ June 2022. Relevant studies were identified using electronic database searches of PubMed, Embase, and PsychInfo, as well as the citation lists of any provisionally included material, and in addition to the studies which cited them. The following search terms were used: depression, major depressive disorder, major depressive episode, depressive symptoms, bipolar, bipolar disorder, mania, manic symptoms, hypomania, hypomanic symptoms, anxiety, anxiety disorder, LGBTIQIA+, LGBTIQ+, LGBT+, LGBT, homosexual, lesbian, gay, bisexual, transgender, transsexual, queer, intersex, and asexual, pansexual, prevalence, experience. The search string used is available in supplementary materials (**Supplementary Table 2**). As homosexuality had been declassified as an independent mental disorder in both the DSM and ICD by 1990, only studies published between 1990 and 23rd June 2022 were extracted. In addition, conference proceedings from 2012–2022 were screened for relevance from the following sources: British Psychological Society, Royal College of Psychiatrists, American Psychiatric Association, Institute for Sexual and Gender Minority Health and Wellbeing, American Psychological Association, Association of LGBTIQ Psychiatrists, Stonewall, World Health Organization, International Society for Affective Disorders, and International Society for Bipolar Disorders.

SJ and OP independently identified and compiled articles identified in searches using Rayyan (13). De-duplication was conducted separately by each researcher, first by using Rayyan’s de-duplication function and then by the reviewers manually screening articles. After de-duplication, titles and abstracts were evaluated for inclusion using the predetermined criteria. Disagreements were discussed and resolved through consensus.

**Figure 1:**
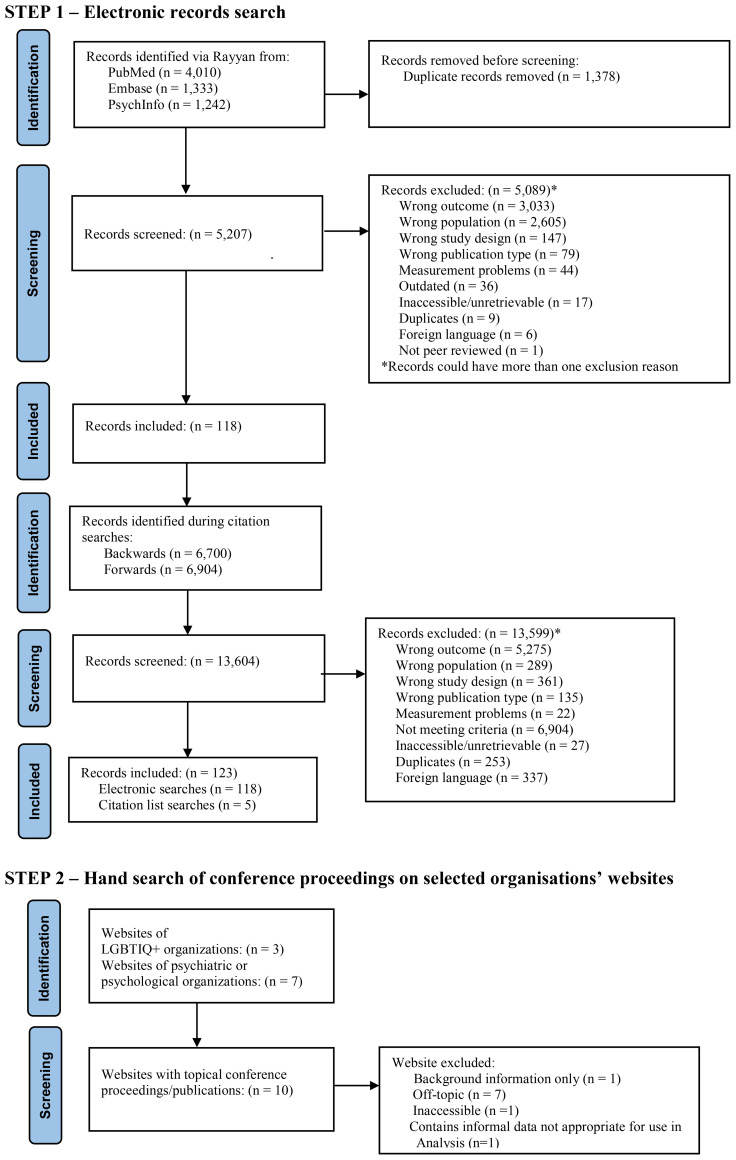
PRISMA 2020 flow diagram for identification and inclusion of the studies.

Reference lists of provisionally included studies were reviewed for relevant parent studies. Additionally, records that had cited the included studies were reviewed for relevance. Researchers then screened abstracts of any selected articles discovered during forwards or backwards searches. The reviewers collaborated and finalized the inclusion list together (**Figure 1**).

### Data collection

Data extraction was performed by SJ and OP, compiling the following information: title, first author, date of publication, research design, sample size, participant selection method, NIH quality rating, age range of participants, country of participant origin, participant gender identity, participant sexual identity, number of participants with depressive, bipolar, and/or anxiety disorders, the proportion of participants with depressive, bipolar, and/or anxiety disorders, the implemented diagnostic criteria, severity of disorder(s), presence (or lack thereof) of psychosis, scores of psychiatric tests, and any additional notes. SJ checked the accuracy of extracted data, manually calculating missing data where possible. Extracted data was collated into Microsoft Excel and are summarised in **Supplementary Table 3**. Studies with missing or derived data are shown in **Supplementary Table 4** and solutions employed in **Supplementary Table 5**.

Societal acceptance of LGBTIQ+ people, measured using the GAI (10), was identified for each country that provided prevalence data. Comparative general population prevalence figures were sourced from Global Disease Burden research (14).

### Study risk of bias assessment

The National Institute of Health (NIH) study quality assessment checklist (15) was used to assess the risk of bias in studies. OP and SJ evaluated each included study using 14 criteria to assign an overall rating of ‘good’, ‘fair’, or ‘poor’ for each study. The two raters completed the checklist independently before comparing results. The NIH study quality assessment checklist questions used can be found in **Supplementary Table 6**.

### Statistical analysis

Sexual and gender identities were assessed at the nominal categorical level. Prevalence rates were assessed as numerical continuous data, considered as the number or proportion of participants with a disorder, expressed as a percentage. If no percentage was reported, it was calculated manually from available data. Confidence intervals of 95% were used in all analyses and where possible, bootstrapping was performed at 1,000 samples. SPSS Version 28.0.1.1 was used for all analysis tasks.

### Ethical considerations

No ethical consideration or approval was required as this was a systematic review of the published evidence base.

## Results

### Study selection

**Figure 1** details the PRISMA flow diagram for the identification and inclusion of the studies included in this review and the PRISMA checklist can be found in supplementary materials (**Supplementary Table 1**). 123 studies (16–138) were included in the review, representing data from 31 countries. 110 studies were used in analyses of the prevalence of depressive disorders, 22 in analyses of bipolar disorders, and 77 in analyses of anxiety disorders. A description of the studies included in this systematic review, including the quality assessment, their geographical location, sample size and LGBTIQ+ population investigated, can be found in **Supplementary Table 3**.

### Worldwide prevalence of mood and anxiety disorders in LGBTIQ+ people

We found that LGBTIQ+ individuals experience depressive disorders at a mean rate of 35.3%, bipolar disorders at a mean rate of 5.6%, and anxiety disorders at a mean rate of 34.3% (**Table 1**). These rates are representative of the prevalence of mood and anxiety disorders in individuals of any combination of gender and sexual identities. Prevalence rates were 8–11 times higher than those reported in the general population (see **Table 1**). Meta-analyses were not conducted due to differences in study methodologies and a noted lack of use of comparator group in selected studies.

**Table 1:** Worldwide prevalence of affective disorders in LGBTIQ+ populations compared to the general population.

Disorder	LGBTIQ+ Mean % (SD, Range at 95% CI)	General population mean %*
Depressive disorders	35.3% (sd = 18.5, 98.8) n = 106 studies	3.8%
Bipolar disorders	5.6% (sd = 5.5, 23.6) n = 22 studies	0.5%
Anxiety disorders	34.3% (sd = 20.2, 90) n = 45 studies	4.1%

*Global Disease Burden (IHME, 2019)

### Regional prevalence of mood and anxiety disorders in LGBTIQ+ people

Prevalence rates of mood and anxiety disorders in LGBTIQ+ people varied depending on continent (see **Table 2**). Where multiple studies were available, the highest prevalence rates of depressive disorders in LGBTIQ+ populations were in Asia, the highest prevalence rates of bipolar disorders in LGBTIQ+ populations were in North America, and the highest prevalence rates of anxiety disorders in LGBTIQ+ populations were in South America. There were relatively few studies investigating the prevalence rates of mood and anxiety disorders in LGBTIQ+ people published from African or South American countries.

**Table 2:** Regional prevalence of affective disorders in LGBTIQ+ populations compared to the general population.

Region	LGBTIQ+ depressive disorders mean % (SD, 95% CI) n	General population depressive disorders mean %	LGBTIQ+ bipolar disorders mean % (SD, 95% CI) n	General population bipolar disorders mean %	LGBTIQ+ anxiety disorders mean % (SD, 95% CI), n	General population anxiety disorders mean %
Africa	24.9% (sd = 15.8, 34.9) n = 6	3.6%	4% (sd = 0, 0) n = 1	0.54%	44% (sd = 0, 0) n = 1	3.52%
Asia	38.2% (sd = 21.3, 98.8) n = 18	3.6%	1.5% (sd = 1.7, 4.2) n = 6	0.38%	26.5% (sd = 21.2, 66.7) n = 12	3.55%
Australasia	38.1% (sd = 16.9, 64.9) n = 32	4.9%	3.3% (sd = 0, 0) n = 1	1.34%	31.3% (sd = 17.1, 64.2) n = 20	6.64%
Europe	26.6% (sd = 21.8, 73.5) n = 25	4.5%	2.5% (sd = 3.5, 8.5) n = 5	0.88%	31.3% (sd = 19.3, 67.5) n = 24	5.15%
North America	36.5% (sd = 17.7, 80) n = 130	4.1%*	7.8% (sd = 5.8, 21.4) n = 20	*0.94%	36.5% (sd = 21.1, 86.9) n = 80	6.12%*
South America	27.9% (sd = 12.7, 27.1) n = 4	4.1%*	N/A n = 0	*0.94%	40.9% (sd = 21.8, 39.5) n = 3	6.12%*

*IHME figures are representative of the Americas as a whole.

### Prevalence by LGBTIQ+ subgroup

This analysis investigated the prevalence of affective disorders in populations of lesbians, gay males, bisexuals (females and males), transgender people (both binary and nonbinary-identified individuals) and males who have sex with males (see **Table 3**). Results of the analysis indicated the highest prevalence of both depressive and anxiety disorders was in transgender people. Bisexual people were found to have the highest prevalence of bipolar disorders, but this finding may be affected by how few studies exist and the small number of participants in the available studies. Conversely, males who have sex with males were found to have the lowest prevalence of both depressive and anxiety disorders, while lesbian females the lowest prevalence of bipolar disorders.

**Table 3:** Worldwide prevalence rates of affective disorders in specific LGBTIQ+ populations.

Identity	Depressive disorders mean % (SD, 95% CI) n	Bipolar disorders mean % (SD, 95% CI) n	Anxiety disorders mean % (SD, 95% CI) n
Lesbian females	35.3% (sd = 12.5, 37.6) n = 13	2.3% (sd = 0, 0) n = 1	31.1% (sd = 24.5, 70.8) n = 8
Gay males	28.1% (sd = 10.4, 42.4) n = 16	8.5% (sd = 0, 0) n = 1	31.1% (sd = 14.4, 36) n = 8
Bisexual males or females	36% (sd = 13, 58.1) n = 33	14.4% (sd = 13,18.4) n = 1	33.7% (sd = 20.5, 79.6) n = 18
Transgender	41% (sd = 23.1, 98.8) n = 67	4% (sd = 3.8, 14.3) n = 5	40.1% (sd = 22.2, 90) n = 65
Males who have sex with males	25.2% (sd = 13.2, 36) n = 14	3.2% (sd = 1.1, 1.6) n = 2	29.8% (sd = 10.5, 23.1) n = 5

### Relationship between prevalence rates and GAI scores

Taking together the studies included in this review from a variety of countries and regions, we did not find a statistically significant relationship between the prevalence of mood and anxiety disorders globally and GAI (10) equality/acceptance ratings (see **Table 4**). Examining this relationship on a regional level, there was a significant correlation in North American LGBTIQ+ populations between GAI (10) equality/acceptance ratings and the prevalence of depressive (r_s_=0.3 p <0.001) and anxiety disorders (r_s_=0.26 p <0.017) but no significant correlations in other regions (see **Table 5**).

**Table 4:** Correlations between worldwide prevalence rates of affective disorders in LGBTIQ+ populations and GAI* acceptance/equality scores.

Disorder	Test conducted	Correlation	Significance	Statistically significant?
Depressive disorders	Spearman’s	0.031	0.646	No
Bipolar disorders	Spearman’s	0.110	0.543	No
Anxiety disorders	Spearman’s	0.043	0.612	No

p ≤ 0.05 indicates statistical significance; *GAI (Williams Institute, 2021)

**Table 5:** Correlations between regional rates of affective disorders in LGBTIQ+ populations and GAI* acceptance/equality scores.

Region	Disorder category, n	Correlation	Significance	Statistically significant?
Africa	Depressive, n = 6	0.174	0.742	No
	Bipolar, n = 1	cannot be calculated	–	–
	Anxiety, n = 1	cannot be calculated	–	–
Asia	Depressive, n = 18	0.023	0.927	No
	Bipolar, n = 6	0.137	0.795	No
	Anxiety, n = 12	0.471	0.123	No
Australasia	Depressive, n = 32	-0.003	0.985	No
	Bipolar, n = 1	cannot be calculated	–	–
	Anxiety, n = 20	0.088	0.711	No
Europe	Depressive, n = 25	-0.390	0.054	No
	Bipolar, n = 5	0.354	0.559	No
	Anxiety, n = 24	-0.19	0.374	No
North America	Depressive, n =130	0.300	<0.001	Yes
	Bipolar, n = 20	-0.055	0.818	No
	Anxiety, n = 80	0.265	0.017	Yes
South America	Depressive, n = 4	0.775	0.225	No
	Bipolar, n = 0	cannot be calculated	–	–
	Anxiety, n = 3	cannot be calculated	–	–

p ≤ 0.05 indicates statistical significance; *GAI (Williams Institute, 2021)

### Quality assessment of included studies

116 studies were rated as ‘good’ and seven were assessed as ‘fair’. No studies were rated as ‘poor’ quality. These are shown in **Supplementary Table 3**.

## Discussion

In this systematic review, we found markedly higher prevalence rates of mood and anxiety disorders in LGBTIQ+ people compared to the overall general population. Globally, we found that LGBTIQ+ people experienced an 8-11-fold higher prevalence rate of depressive disorders, bipolar disorders and anxiety disorders compared to the general population. This pattern of increased prevalence occurred irrespective of continent. We found that rates of depressive disorders in LGBTIQ+ populations were 6–11 times higher across continents. In continents where multiple studies were available, rates of bipolar disorders in Asian, European and North American LGBTIQ+ populations were 3–8 times higher, and rates of anxiety disorders in Asian, Australasian, European, North and South American LGBTIQ+ populations were 5–7 times higher. These findings need to be considered in the context of over-representation of studies from North America, and a relative lack of studies from Africa and South America, together with the under representation of certain diagnostic categories and sexual identities.

There did not seem to be a great difference in the prevalence rates of depressive or anxiety disorders between LGBTIQ+ communities, for example, between gay males and lesbian females, although rates did seem to be comparatively higher in transgender people. There was more variation in the prevalence rates of bipolar disorders across LGBTIQ+ groups, which may reflect the small number of studies available.

Given how common lived experiences of homophobia, heteronormativity, rejection by family and friends, discrimination, and victimization due to LGBTIQ+ status are (7–9, 19) higher prevalence rates of depressive (35.5% v. 3.8%), bipolar (5.6% v. 0.5%) and anxiety (34.3% v. 4.1%) disorders were not unexpected. However, we were surprised at how much higher the prevalence rates we found in our review were compared to the general population. This is an important finding and underlines the public health importance of providing LGBTIQ+ informed mental healthcare to LGBTIQ+ people with, and at risk for developing, mood and anxiety disorders.

The correlations between Global Acceptance Index scores (10) and depressive and anxiety disorders in North America were the only statistically significant correlations found, and they were unexpectedly positive. This positive correlation might indicate that relative social acceptance, the metric measured by the GAI, is not a large contributor to the diagnosis of depressive and/or anxiety disorders in LGBTIQ+ individuals outside of North America. Further examination into alternative factors for the development of mood disorders in LGBTIQ+ people is needed. With that said, because the North American continent was so over-represented relative to other regions in these analyses, the results found for North America may be more sensitive than those for other, less represented areas.

Overall, all studies included in the review, regardless of their country of origin, were of good or fair quality.

### Strengths and limitations

The strengths of this systematic review include the availability of research studies for inclusion in analysis, the wide geographical spread of these studies and the representation of transgender people in the research identified. High quality research was found to be available from all six inhabited continents, making the review worldwide in scope. Moreover, changing attitudes about sexual and gender minority identity, as well as mental health conditions, have allowed more people to participate openly in this kind of research. Transgender individuals, and their mental health, have been studied extensively as of late, offering a range of information upon which to build statistical analyses.

Along with strengths, there are a number of limitations to consider. A limitation of the evidence base was that there was a comparative lack of studies of mood and anxiety disorder prevalence rates in gay males and lesbian females compared to bisexual and transgender people. We did not identify any studies investigating mood and anxiety prevalence rates in participants with less common identities, including pansexual, omnisexual, demisexual, asexual, nonbinary, genderqueer/genderfluid, and agender (15). Such individuals are minorities in the LGBTIQ+ population and merging diverse identities into a single LGBTIQ+ category may mask mental health challenges unique to specific LGBTIQ+ identities. There was also a relative lack of studies investigating the prevalence of bipolar disorders in LGBTIQ+ people, and the prevalence of affective disorders in LGBTIQ+ populations in Africa and South America. The severity of mood and anxiety disorders was also not measured by most studies and so the degree of impairment associated with these disorders experienced by LGBTIQ+ people is hard to assess.

Limitations of the review were that only studies published in, or translated to, English were included; and that some of the included studies did not necessarily report the period over which they measured prevalence rates and so we could not specify our prevalence estimates to a specific time periods.

### Implications and future research

This systematic review highlights the continuing need for sexual and/or gender-identity affirmative mental healthcare worldwide to tackle the higher prevalence rates of affective disorders we identified in LGBTIQ+ people. Affirmative and culturally competent mental healthcare could be key in the improvement and protection of mental health in LGBTIQ+ people, possibly serving as protective factors against both environmental and internal experiences of homophobia, heteronormativity and stigma.

We would suggest that future research particularly investigates the prevalence of bipolar disorders in LGBTIQ+ people as there is a relative lack of research in this area. Furthermore, as current research has tended towards examining mood and anxiety disorder prevalence rates in bisexual and transgender people, it would be beneficial to conduct further studies focusing on prevalence rates in lesbian females and gay males in order to provide an updated comparison. Finally, more research is needed exploring the prevalence of mood and anxiety disorders in less common LGBTIQ+ identities.

## Conclusions

We identified that mood and anxiety disorder prevalence rates are 8- to 11-fold higher in LGBTIQ+ people compared to the general population, indicating the need for better preventative mental health interventions. We found a relative lack of studies in bipolar disorders, gay males, lesbian females and other individuals identifying as LGBTIQ+, and in LGBTIQ+ populations in Africa and South America. Our study suggests that the proactive prevention and treatment of mood and anxiety disorders in LGBTIQ+ populations is important and calls for further work exploring factors influencing prevalence rates of affective disorders in LGBTIQ+ communities.

## Data Availability

The original contributions presented in the study are included in the article/supplementary material. Further inquiries can be directed to the corresponding author.
