# Supplementary material for: Exploring the global prevalence of mood and anxiety disorders in LGBTIQ+ people: A systematic review
Source: Front Psychiatry. 2025 Dec 4;16:1662265. doi: 10.3389/fpsyt.2025.1662265 (PMC12711858; doi:10.3389/fpsyt.2025.1662265)
Supplement: Supplementary file 1 [file Supplementaryfile1.docx]

**Supplementary Materials**

Table of Contents

[Supplementary Table 1. PRISMA 2020 checklist 2](#_Toc210927927)

[Supplementary Table 2. Electronic database search strategy 6](#_Toc210927928)

[Supplementary Table 3. Study characteristics 8](#_Toc210927929)

[Supplementary Table 4. Studies with missing or derived data 13](#_Toc210927930)

[Supplementary Table 5. Studies with potential data issues and solutions employed 14](#_Toc210927931)

[Supplementary Table 6. NIH STUDY QUALITY ASSESSMENT QUESTIONS 16](#_Toc210927932)

# Supplementary Table 1. PRISMA 2020 checklist

| **Section and Topic** | **Item #** | **Checklist item** | **Location where item is reported** |
| --- | --- | --- | --- |
| **TITLE** | | |  |
| Title | 1 | Identify the report as a systematic review. | Title |
| **ABSTRACT** | | |  |
| Abstract | 2 | See the PRISMA 2020 for Abstracts checklist. | Abstracts checklist |
| **INTRODUCTION** | | |  |
| Rationale | 3 | Describe the rationale for the review in the context of existing knowledge. | Abstract; Introduction |
| Objectives | 4 | Provide an explicit statement of the objective(s) or question(s) the review addresses. | Abstract; Introduction |
| **METHODS** | | |  |
| Eligibility criteria | 5 | Specify the inclusion and exclusion criteria for the review and how studies were grouped for the syntheses. | Methods – eligibility criteria |
| Information sources | 6 | Specify all databases, registers, websites, organisations, reference lists and other sources searched or consulted to identify studies. Specify the date when each source was last searched or consulted. | Methods – Information Sources |
| Search strategy | 7 | Present the full search strategies for all databases, registers and websites, including any filters and limits used. | Methods – Search Strategy |
| Selection process | 8 | Specify the methods used to decide whether a study met the inclusion criteria of the review, including how many reviewers screened each record and each report retrieved, whether they worked independently, and if applicable, details of automation tools used in the process. | Methods – Selection process |
| Data collection process | 9 | Specify the methods used to collect data from reports, including how many reviewers collected data from each report, whether they worked independently, any processes for obtaining or confirming data from study investigators, and if applicable, details of automation tools used in the process. | Methods – Data collection |
| Data items | 10a | List and define all outcomes for which data were sought. Specify whether all results that were compatible with each outcome domain in each study were sought (e.g. for all measures, time points, analyses), and if not, the methods used to decide which results to collect. | Methods – Data collection |
|  | 10b | List and define all other variables for which data were sought (e.g. participant and intervention characteristics, funding sources). Describe any assumptions made about any missing or unclear information. | Methods – Data collection |
| Study risk of bias assessment | 11 | Specify the methods used to assess risk of bias in the included studies, including details of the tool(s) used, how many reviewers assessed each study and whether they worked independently, and if applicable, details of automation tools used in the process. | Methods – Study risk of bias assessment |
| Effect measures | 12 | Specify for each outcome the effect measure(s) (e.g. risk ratio, mean difference) used in the synthesis or presentation of results. | Methods – Statistical analysis |
| Synthesis methods | 13a | Describe the processes used to decide which studies were eligible for each synthesis (e.g. tabulating the study intervention characteristics and comparing against the planned groups for each synthesis (item #5)). | Methods – Data collection and Supplementary Table 1 |
|  | 13b | Describe any methods required to prepare the data for presentation or synthesis, such as handling of missing summary statistics, or data conversions. | Methods – Statistical analysis |
|  | 13c | Describe any methods used to tabulate or visually display results of individual studies and syntheses. | Supplementary Table 1; Tables 1-5 |
|  | 13d | Describe any methods used to synthesize results and provide a rationale for the choice(s). If meta-analysis was performed, describe the model(s), method(s) to identify the presence and extent of statistical heterogeneity, and software package(s) used. | Methods – Statistical analysis |
|  | 13e | Describe any methods used to explore possible causes of heterogeneity among study results (e.g. subgroup analysis, meta-regression). | N/A |
|  | 13f | Describe any sensitivity analyses conducted to assess robustness of the synthesized results. | N/A |
| Reporting bias assessment | 14 | Describe any methods used to assess risk of bias due to missing results in a synthesis (arising from reporting biases). | Supplemental material – Missing/Derived data and Assumptions and potential data problems |
| Certainty assessment | 15 | Describe any methods used to assess certainty (or confidence) in the body of evidence for an outcome. | Methods – Statistical analysis (confidence intervals) |
| **RESULTS** | | |  |
| Study selection | 16a | Describe the results of the search and selection process, from the number of records identified in the search to the number of studies included in the review, ideally using a flow diagram. | Results – Study selection |
|  | 16b | Cite studies that might appear to meet the inclusion criteria, but which were excluded, and explain why they were excluded. | Figure 1 – PRISMA chart |
| Study characteristics | 17 | Cite each included study and present its characteristics. | Supplementary Table 1 |
| Risk of bias in studies | 18 | Present assessments of risk of bias for each included study. | Results |
| Results of individual studies | 19 | For all outcomes, present, for each study: (a) summary statistics for each group (where appropriate) and (b) an effect estimate and its precision (e.g. confidence/credible interval), ideally using structured tables or plots. | Supplementary Table 1 |
| Results of syntheses | 20a | For each synthesis, briefly summarise the characteristics and risk of bias among contributing studies. | Results and Supplementary Materials |
|  | 20b | Present results of all statistical syntheses conducted. If meta-analysis was done, present for each the summary estimate and its precision (e.g. confidence/credible interval) and measures of statistical heterogeneity. If comparing groups, describe the direction of the effect. | Results, Tables 1-5 |
|  | 20c | Present results of all investigations of possible causes of heterogeneity among study results. | N/A |
|  | 20d | Present results of all sensitivity analyses conducted to assess the robustness of the synthesized results. | N/A |
| Reporting biases | 21 | Present assessments of risk of bias due to missing results (arising from reporting biases) for each synthesis assessed. | Supplementary materials and Supplementary Table 2 |
| Certainty of evidence | 22 | Present assessments of certainty (or confidence) in the body of evidence for each outcome assessed. | Supplementary materials |
| **DISCUSSION** | | |  |
| Discussion | 23a | Provide a general interpretation of the results in the context of other evidence. | Discussion |
|  | 23b | Discuss any limitations of the evidence included in the review. | Discussion – Strengths and limitations |
|  | 23c | Discuss any limitations of the review processes used. | Discussion – Strengths and limitations |
|  | 23d | Discuss implications of the results for practice, policy, and future research. | Discussion – Implications and future research |
| **OTHER INFORMATION** | | |  |
| Registration and protocol | 24a | Provide registration information for the review, including register name and registration number, or state that the review was not registered. | Abstract and Methods – Protocol |
|  | 24b | Indicate where the review protocol can be accessed, or state that a protocol was not prepared. | Abstract and Methods – Protocol |
|  | 24c | Describe and explain any amendments to information provided at registration or in the protocol. | N/A |
| Support | 25 | Describe sources of financial or non-financial support for the review, and the role of the funders or sponsors in the review. | Funding statement |
| Competing interests | 26 | Declare any competing interests of review authors. | Declaration of Interest |
| Availability of data, code and other materials | 27 | Report which of the following are publicly available and where they can be found: template data collection forms; data extracted from included studies; data used for all analyses; analytic code; any other materials used in the review. | Data availability |

# Supplementary Table 2. Electronic database search strategy

| Order | Search terms |
| --- | --- |
| 1 | Depression |
| 2 | major depressive disorder |
| 3 | major depressive episode |
| 4 | depressive symptoms |
| 5 | Bipolar |
| 6 | bipolar disorder |
| 7 | Mania |
| 8 | manic symptoms |
| 9 | Hypomania |
| 10 | hypomanic symptoms |
| 11 | Anxiety |
| 12 | anxiety disorder |
| 13 | panic disorder |
| 14 | generalized anxiety disorder |
| 15 | GAD |
| 16 | social anxiety disorder |
| 17 | OR / 1-16 |

| Order | Search terms |
| --- | --- |
| 18 | LGBTQIA+ |
| 19 | LGBTIQ+ |
| 20 | LGBT+ |
| 21 | Homosexual |
| 22 | Lesbian |
| 23 | Gay |
| 24 | Bisexual |
| 25 | Transgender |
| 26 | Transsexual |
| 27 | Queer |
| 28 | Intersex |
| 29 | Asexual |
| 30 | Pansexual |
| 31 | OR / 18-30 |

| Order | Search terms |
| --- | --- |
| 32 | Prevalence |
| 33 | Experience |
| 34 | OR / 32-33 |
| 35 | #17 AND #31 AND #34 |

Supplementary Table 3. Study characteristics

# Supplementary Table 4. Studies with missing or derived data

| **Missing/Derived Data** | | |
| --- | --- | --- |
| ***Data issue*** | ***Studies*** | ***Solution employed*** |
| Missing prevalence proportions | Hoshiai, et al., 2010  Veale, et al., 2017 | calculated by dividing the number of participants by the number of affected individuals |
| Missing prevalence proportion totals | Galea, et al., 2021 Jankovic, et al., 2020 | calculated by finding the sum of participants with PHQ-9 scores above the conventional cutoff score of 10 and deriving the proportion |
| Data split by race with no totals | Lopez, et al., 2021 | calculated by finding the sums of split data |
| Data split by specific disorder | Kerridge, et al., 2017  Meybodi, et al., 2014 | calculated by finding sum totals of disorder categories: depressive disorders, bipolar disorders, anxiety disorders |
| Data split by hormone treatment status | Gomez-Gil, et al., 2012 | calculated by finding the sums of split data |
| Double diagnosis | Rogers, et al., 2003 | calculated by separating “double diagnoses” of major depressive disorder and persistent depressive disorder from diagnosis of one or the other |

# Supplementary Table 5. Studies with potential data issues and solutions employed

| ***Data issue*** | ***Studies*** | ***Solution employed*** |
| --- | --- | --- |
| Gender identity not specified in studies with sexual minority identities (ie LBG+) | Ahaneku, et al., 2016  Barnhill, et al., 2017  Bjorkenstam, et al., 2017  Blashill & Calzo, 2019  Bolton & Sareen, 2011  Bostwick, et al., 2010  Bostwick, et al., 2015  Burns, et al., 2015  Chaudhry & Reisner, 2019  Cochran, et al., 2007  Cochran & Mays, 2009  Fergusson, et al., 1999  Gilman, et al., 2001  Grabovac, et al., 2019  Jankovic, et al., 2020  Kerridge, et al., 2017  Kipke, et al., 2007  Kunzweiler, et al., 2018  Mao, et al., 2009  McGarty, et al., 2021  Mills, et al., 2004  Oginni, et al., 2018  Oswal, et al., 2017  Prestage, et al., 2018  Renteria, et al., 2020  Rogers, G., et al., 2003  Secor, et al., 2015  Spittlehouse, et al., 2020  Stahlman, et al., 2015  Steele, et al., 2009  Stoloff, et al., 2013  Thirunavukkarasu, et al., 2021  Wang, et al., 2007  Yi, et al., 2017  Yu, et al., 2013  Zietsch, et al., 2012 | Assumed gender identity to be cisgender as it is considered normative and gender identities other than cis are usually stipulated |
| Disorders labeled as “mood disorders” | Blashill & Calzo, 2019  Bolton & Sareen, 2011  Heylens, et al., 2014  Hyde, et al., 2015  She, et al., 2020 | excluded from analysis due to lack of specificity |
| Prevalence not reported | Liu, et al., 2018  Scott, et al., 2017  Steele, et al., 2009  Warne, et al., 2005 | excluded from analysis |
| Measurement inconsistency | Castelo-Branco, et al., 2021 | excluded from analysis |
| Participants allowed to select multiple gender identities | Parodi, et al., 2022 | excluded from analysis due to lack of specificity |
| Likely typographical error - bipolar disorder prevalence listed at 75% | Nahata, et al., 2017 | excluded from analysis |

# Supplementary Table 6. NIH study quality assessment questions

1. Was the research question or objective in this paper clearly stated?
2. Was the study population clearly specified and defined?
3. Was the participation rate of eligible persons at least 50%?
4. Were all the subjects selected or recruited from the same or similar populations (including the same time period)? Were inclusion and exclusion criteria for being in the study prespecified and applied uniformly to all participants?
5. Did a sample size justification, power description, or variance effect estimates provided?
6. For the analyses in this paper, were the exposure(s) of interest measured prior to the outcome(s) being measured?
7. Was the timeframe sufficient so that one could reasonably expect to see an association between exposure and outcome if it existed?
8. For exposures that can vary in amount or level, did the study examine different levels of the exposure as related to the outcome (eg categories of exposure, or exposure measured as continuous variable)?
9. Were the exposure measures (independent variables) clearly defined, valid, reliable, and implemented consistently across all study participants?
10. Was the exposure(s) assessed more than once over time?
11. Were the outcome measures (dependent variables) clearly defined, valid, reliable, and implemented consistently across all study participants?
12. Were the outcome assessors blinded to the exposure status of participants?
13. Was loss to follow-up after baseline 20% or less?
14. Were key potential confounding variables measured and adjusted statistically for their impact on the relationship between exposure(s) and outcome(s)?
